# Heparin-Binding Hemagglutinin Adhesin (HBHA) Is Involved in Intracytosolic Lipid Inclusions Formation in Mycobacteria

**DOI:** 10.3389/fmicb.2018.02258

**Published:** 2018-09-24

**Authors:** Dominique Raze, Claudie Verwaerde, Gaspard Deloison, Elisabeth Werkmeister, Baptiste Coupin, Marc Loyens, Priscille Brodin, Carine Rouanet, Camille Locht

**Affiliations:** CNRS UMR8204, INSERM U1019, Centre d’Infection et d’Immunité de Lille, Institut Pasteur de Lille, Université de Lille, Lille, France

**Keywords:** tuberculosis, *Mycobacterium*, lipids, dormancy, carbon storage

## Abstract

The heparin-binding hemagglutinin adhesin (HBHA) is an important virulence factor of *Mycobacterium tuberculosis*. It is a surface-displayed protein that serves as an adhesin for non-phagocytic cells and is involved in extra-pulmonary dissemination of the tubercle bacillus. It is also an important latency antigen useful for the diagnosis of latently *M. tuberculosis*-infected individuals. Using fluorescence time-lapse microscopy on mycobacteria that produce HBHA-green fluorescent protein chimera, we show here that HBHA can be found at two different locations and dynamically alternates between the mycobacterial surface and the interior of the cell, where it participates in the formation of intracytosolic lipid inclusions (ILI). Compared to HBHA-producing mycobacteria, HBHA-deficient mutants contain significantly lower amounts of ILI when grown *in vitro* or within macrophages, and the sizes of their ILI are significantly smaller. Lipid-binding assays indicate that HBHA is able to specifically bind to phosphatidylinositol and in particular to 4,5 di-phosphorylated phosphatidylinositol, but not to neutral lipids, the main constituents of ILI. HBHA derivatives lacking the C-terminal methylated, lysine-rich repeat region fail to bind to these lipids and these derivatives also fail to complement the phenotype of HBHA-deficient mutants. These studies indicate that HBHA is a moonlighting protein that serves several functions depending on its location. When surface exposed, HBHA serves as an adhesin, and when intracellularly localized, it participates in the generation of ILI, possibly as a cargo to transport phospholipids from the plasma membrane to the ILI in the process of being formed.

## Introduction

Tuberculosis remains one of the major global public health threats. The main causative agent, *Mycobacterium tuberculosis*, is able to adopt multiple strategies to escape from the immune system, to persist silently within cells and to reactivate (Peddireddy et al., 2017). This life cycle within the host relies on a major step: the dormancy of the bacteria leading to latency of the disease. Dormancy is a strategy exploited by many organisms to resist harsh environmental conditions, including nutrient starvation, acidic stress and oxygen limitation. These conditions often lead to a metabolic switch toward the utilization of fatty acids rather than carbohydrates. During stress, actinobacteria, including mycobacteria, as well as some Gram-negative bacteria and cyanobacteria, usually accumulate triacylglycerol (TAG) or wax esters in the form of granules within the cytoplasm (Alvarez and Steinbuchel, 2002).

Mycobacterial dormancy has been studied using several *in vitro* models mimicking various stress conditions. This has led to the discovery of genes upregulated in these conditions. Among them, the gene encoding the heparin-binding hemagglutinin adhesin (HBHA), a major adhesin of *M. tuberculosis*, has been shown to be upregulated in hypoxic conditions (Iona et al., 2016). HBHA is a 198-residue, intrinsically disordered protein (IDP) organized in four distinct domains: an N-terminal short hydrophobic sequence, a coiled-coil domain, a linker domain and a C-terminal heparin-binding domain that is mainly composed of alanine, lysine, and proline residues (Menozzi et al., 1998; Delogu and Brennan, 1999; Lebrun et al., 2012). This latter domain undergoes post-translational methylation of the lysine residues (Pethe et al., 2002). HBHA is present at the outermost layer of the bacterial cell, mediates the attachment of the bacilli to non-phagocytic cells, such as epithelial cells and fibroblasts, induces mycobacterial aggregation (Menozzi et al., 1996) and is involved in extrapulmonary dissemination of *M. tuberculosis* (Pethe et al., 2001). It is also considered as an important marker of latency, as evidenced by a strong interferon gamma response to HBHA in latently infected subjects, as opposed to patients with active tuberculosis (Hougardy et al., 2007). Finally, it has been shown to be protective in murine challenge models (Parra et al., 2004; Temmerman et al., 2004), provided it is combined with the appropriate adjuvant (Verwaerde et al., 2014), and, when used as a booster vaccine, to significantly enhance protection induced by Bacillus Calmette–Guérin (BCG) (Rouanet et al., 2009), the only vaccine currently available against tuberculosis. Among actinobacteria, some *Rhodococcus* species are TAG-accumulating specialists and proteins with HBHA homology have been shown to be involved in this process (MacEachran et al., 2010; Ding et al., 2012).

In this study, we used various imaging technologies to examine the localization of HBHA in the mycobacteria. Previous studies using electron microscopy (Menozzi et al., 1996) and atomic force microscopy (AFM) (Dupres et al., 2005) have shown HBHA at the bacterial cell surface. By using fluorescence time-lapse microscopy, we describe here two different localizations of HBHA that can alternate dynamically from the surface to the cytoplasm during the growth of the bacteria. Intracellular HBHA was found to be associated with neutral lipid vesicles accumulated during stress conditions, which were much less abundant in a HBHA-deficient mutant. We also highlight specific interactions between HBHA and (4,5) di-phosphorylated phosphatidylinositol, an important constituent of membranes and found on surface of lipid vesicles in eukaryotes (Li et al., 2008). All these observations indicate a role of HBHA in the formation of intracytosolic lipid inclusions (ILI).

## Materials and Methods

### Bacterial Strains and Growth Media

The bacterial strains used in this study are listed in **Supplementary Table S1**. *Escherichia coli* strains used for cloning and plasmid propagation were grown on solid or in liquid Luria-Bertani medium supplemented with the appropriate antibiotics. The mycobacterial strains were grown at 37°C in Middlebrook 7H9 supplemented with Middlebrook oleic acid albumin dextrose catalase enrichment (OADC; BD Biosciences) and 0.05% tyloxapol or in Sauton’s medium. Growth on solid medium was performed on 7H10 agar plates supplemented with OADC for 21 days at 37°C. When required, kanamycin and hygromycin were added to the medium at 25 and 50 μg/ml, respectively. *M. tuberculosis* was manipulated in a BSL3 laboratory. *Rhodococcus opacus* PD630 was obtained from Leibniz Institute DSMZ (Germany) and was grown in mineral salt medium (MSM) supplemented with 4% glucose and 0.15% ammonium sulfate (Alvarez et al., 1996).

### DNA Manipulations

Restriction enzyme digests, cloning, subcloning and DNA electrophoresis were done according to standard techniques (Sambrook and Russell, 1989). Primers were synthesized by Eurogentec (Liège, Belgium) and are listed in **Supplementary Table S1**. All PCR were performed by using Hot Star Taq (Qiagen), and the amplicons were first inserted into pCR2.1-TOPO (ThermoFisher Scientific) and then sequenced (Genoscreen, Lille, France). The inserts were subcloned by using FastDigest restriction enzymes (ThermoFisher Scientific). Ligations were performed by using Fast-link DNA ligase (Epicentre, Tebu). Plasmid DNA was prepared by using the QIAprep Spin Miniprep Kit (Qiagen) as recommended by the manufacturer. DNA fragments were purified by using either the QIAquick Gel Extraction Kit or the QIAquick PCR Purification Kit (Qiagen).

### Plasmid Construction

To construct the expression vector pSD-HBHA, the *hbhA* gene was amplified by using pKK-HBHA (Pethe et al., 2001) as template and primers SPhbhA pSD and ASPhbhA pSD, the latter containing a stop codon upstream of the EcoRV site. The 617-bp amplicon was then digested by BamHI and EcoRV and inserted into the same sites of pSD26 (Daugelat et al., 2003), generating pSD-HBHA, which was used to transform *Mycobacterium smegmatis* mc^2^155. To construct the pSD-HBHA-ΔC, a PCR fragment corresponding to the codons for the first 158 amino acids of *hbhA* was amplified by using pKK-HBHA as template and primers SPhbhA pSD and ASPhbhADC pSD. The 497-bp amplicon was then digested by BamHI and EcoRV and inserted into the same sites of pSD26. The resulting plasmid, pSD-HBHA-ΔC, was then introduced into *M. smegmatis* mc^2^155. The production of the expected proteins encoded by pSD-HBHA and pSD-HBHA-ΔC in the recombinant *M. smegmatis* was verified by immunoblotting using the anti-HBHA monoclonal antibody VF2 (see below), which reacts with the linker domain of the protein.

In order to fuse the *egfp* gene to the 3′ end of *hbhA*, a BamHI site was introduced behind the last codon of *hbhA* by PCR using primers SPhbhA Sph Bam and ASPhbhA Sph Bam and pYUB-HBHA (Pethe et al., 2001) as template. The 904-bp amplicon encompassing *hbhA* and its promoter was then digested by SphI and BamHI and inserted into pYUB-HBHA digested by the same enzymes to generate pYUBHB. Amplification of *egfp* was done by using pEGFP-C1 (Clontech) as template and SP/PCR2 egfp1 and ASP/PCR2 egfp1 as primers. The 739-bp PCR fragment was digested by BamHI and PacI and inserted into pYUBHB digested by the same enzymes to generate the fusion between *hbhA* and *egfp*. This plasmid, named pYUBHEGFP, served as template to amplify the entire chimeric open reading frame without the promoter sequence. The amplicon was then digested by EcoRI and HindIII and inserted into the same sites of pMV361 (Stover et al., 1991), downstream of the *hsp60* promoter, thereby generating the pMV361-HE. The kanamycin resistance gene (*kanR*) of this plasmid was not suitable as selective marker for the transformation of kanamycin-resistant BCG*ΔhbhA*. Therefore, *kanR* of pMV361-HE was replaced by the hygromycin resistance gene (*hygR*) with its own promoter sequence. This gene was amplified by using pSD26 (Daugelat et al., 2003) as template and SP HygR and ASP HygR as primers. The 1303-bp amplicon was digested by NheI and SpeI and inserted into the same restriction sites of pMV361-HE, thereby generating the integrative plasmid pMV361-HEH. This construct was inserted into the chromosome of BCG*ΔhbhA* at the *attP* site and the production of the hybrid protein was verified by immunoblot analysis with monoclonal anti-HBHA VF2 and monoclonal anti-Green Fluorescent Protein GF28R (ThermoFisher Scientific). A detailed list and origin of plasmids and primers are presented in **Supplementary Table S1**.

### Generation of the Anti-HBHA Monoclonal Antibody VF2

To immunize mice, native HBHA (nHBHA) was purified by heparin-Sepharose followed by reverse-phase HPLC as described previously (Masungi et al., 2002). Three sub-cutaneous injections at 3-week intervals of 10 μg purified nHBHA in the presence of 150 μg DDA (Sigma) and 25 μg MPL (Sigma) used as adjuvants were done in BALB/c mice (Charles River). Mice producing sera with the highest ELISA titer against nHBHA were selected and boosted with an intra-venous injection of 10 μg purified nHBHA, 3 days before the sacrifice. After sacrifice, the fusion of myelomas SP2/O and spleen cells was done as described by Köhler and Milstein (1975) and hybridomas were selected for the highest ELISA titer against nHBHA. The selected monoclonal antibody VF2 is able to recognize both native HBHA and non-methylated recombinant HBHA.

### Induction of Intracytosolic Lipid Inclusions

A 10-ml of static BCG culture was inoculated at an OD_600nm_ of 0.5 in 7H9-OADC or Sauton’s medium, and after 24 h at 37°C, the culture was diluted at an OD_600nm_ of 0.5 and treated at 37°C for 16 h with the NO donor spermine/NONOate (Enzo Life Sciences; 100 μM final). Then, 500 μl of the culture were centrifuged at 2,300 × *g* and the pellet was resuspended in 1 ml of distilled water for fluorescent dye staining and microscopy. For *M. smegmatis*, the same conditions were used, except that the culture was treated with spermine/NONOate at an OD_600nm_ of 0.2 instead of 0.5.

### Macrophage Cell Culture and *in vitro* Infection

The mouse RAW2647 macrophage cell line (ATCC, United Kingdom) was grown in DMEM containing 5% fetal calf serum (FCS, Gibco, ThermoFisher Sientific) at 37°C under 5% CO_2_. Lack of cell activation was checked before their use by microscopic observation and evidenced by the round phenotype and absence of pseudopods. Bacterial stocks were quantified by measuring the optical density (OD_600nm_) and/or green fluorescent protein (GFP) fluorescence on a Victor Multilabel Counter (Perkin Elmer).

For infection, cells (15×10^3^/well) were seeded in black 96-well plates in DMEM-5% FCS and infected the next day with MT103-GFP or MT103*ΔhbhA*-GFP at a ratio of 10 bacteria/cell for 4 h. Each experimental condition was performed in 10 different wells. Amikacin (200 μg/ml final) was then added for 2 h to remove extracellular bacteria. The cells were washed with DMEM and finally incubated at 37°C for 24 h in DMEM-5% FCS. Cells were then fixed with 4% paraformaldehyde (PFA) for 20 min, washed with DPBS (Gibco, ThermoFisher Scientific) and their nuclei were labeled with a DAPI solution (5 mg/ml final; ThermoFisher Scientific). After washing in DPBS, the neutral lipid content in the cells and the bacteria was revealed by at least 30 min incubation with a solution of HCS LipidTOX Red Neutral Lipid Stain (LipidTOX Red, ThermoFisher Scientific) diluted 1/300. Plates were then read using the high-content screening (HCS) technology.

### Fluorescence Microscopy

For fluorescent lipid staining, two fluorochromes were used: Nile Red (Sigma) for fixed bacteria on slides and LipidTOX Red for living organisms used in HCS or time-lapse microscopy. In the case of Nile Red staining, bacterial solutions in water were layered onto Teflon printed diagnostic slides (Immuno-Cell int.) and fixed by heating at 90°C.

After cooling to room temperature, the slides were covered with a Nile Red solution (100 ng/ml ethanol) for 15 min in the dark. The slides were gently washed twice with water and 1% KMnO_4_ was added for 1 min followed by two washes with water. LipidTOX Red staining was performed directly on liquid cultures by adding the fluorochrome (1/300). After at least 30 min incubation in the dark, the samples were mounted onto glass slides as described for the Nile Red staining above. The mounted slides were observed on a Zeiss Axioplan2 microscope with a 63× or 100× oil immersion lens. The rhodamine filter was used and the images were captured with a CCD camera controlled by MetaVue software from Zeiss.

### Electron Microscopy

The mycobacteria were fixed in 0.1 M phosphate buffer containing 4% PFA for 3 h at room temperature. After washing with PBS, bacterial pellets were suspended in PBS and embedded in 12% gelatin, infiltrated with 2.3 M sucrose over night at 4°C and then cut into small blocks, which were mounted on pins and frozen in liquid nitrogen. Ultrathin cryosections of 70 nm thickness were obtained by cutting at -120°C with an ultra cryomicrotome from Leica. Cryosections were picked up in a 1:1 mixture of 2% methylcellulose and 2.3 M sucrose and laid down on formvar-coated grids at room temperature. Sections were contrasted with a 1:9 mixture of 3% uranyl-acetate and 2% methylcellulose and finally observed with a Hitachi H7500 TEM (Elexience, France) and images were acquired with a one Mpixel digital camera from AMT (Elexience, France).

### Time-Lapse Microscopy

Live cell imaging of BCG was performed as described (Joyce et al., 2011). Briefly, recombinant BCG cultures at early stage of growth (OD_600nm_ between 0.5 and 0.8) were centrifuged and resuspended in water containing 50 μg/ml hygromycin. For ILI staining, the bacterial suspension was incubated with LipidTOX Red (1/300) at 37°C for 25 min in the dark. Six hundred-microliter of the bacterial suspension was then applied onto a 35 mm Nunc glass bottom dish with a diameter of viewing area of 12 mm (ThermoFisher Scientific) and incubated at 37°C for 15 min in the dark. After removing remaining liquid, the samples were air-dried under the hood for 10 min. Then, 3 ml of 0.6% Noble agar (Difco, BD Biosciences) suspended in Sauton’s medium with 50 μg/ml hygromycin and 1/300 LipidTOX Red was carefully added over the bacterial layer. Samples were observed with a Zeiss LSM880 Confocal microscope to obtain high-resolution images (voxel 0.079 × 0.079 × 1 μm^3^) using an Airy scan detector with a ×40 oil immersion lens (EC Plan Neofluar 40×/0.30 oil). Images were processed using ZEN software for Airy scan processing and two sections plane of the Z stack were selected for a maximum projection by choosing for each pixel the highest signal value in any of the sections. Two lasers (488 and 561 nm) were used for excitation of GFP and LipidTOX. The bacteria were imaged at 37°C and 5% CO_2_ with snapshots every 90 min.

### Image Acquisition by Automated Confocal Microscopy

For HCS, images were acquired using an automated fluorescent confocal microscope OPERA (PerkinElmer), with a 63× (NA 1.2) water immersion lens. The microscope was equipped with 405, 488, 561, and 640 nm excitation lasers. The emitted fluorescence was captured using three cameras associated with a set of appropriate filters covering a detection wavelength ranging from 450 to 690nm. Results were collected from at least 10 different wells and with 8–10 acquisitions per well. After acquisition, images from the automated confocal microscope were analyzed using the image-analysis software Acapella 2.6 (PerkinElmer).

### Atomic Force Microscopy

Atomic force microscopy was performed by using heparin-coated tips on BCG, BCG-HEH, and BCG*ΔhbhA* as previously described (Veyron-Churlet et al., 2018).

### Lipid Binding Assays

All lipids and membrane lipid strips were obtained from Echelon Biosciences Inc., (Salt Lake City, UT, United States), except for the *E. coli* Polar Lipid extract, which was purchased from Avanti Polar Lipids, Inc., (Alabaster, AL, United States). Lipid binding assays were first performed using a commercial membrane lipid strip (product number: P-6002). The strips were blocked for 1 h using 3% fat free BSA in 20 mM Tris-HCl (pH 7.5), 150 mM NaCl (TBS). After blocking, the membranes were incubated each with the different HBHA preparations at 0.5 μg/ml in 3% BSA-TBS over night at 4°C. The lipid strips were then washed with TBS containing 0.1% Tween-20 (TBST) and lipid-binding HBHA was detected by incubation for 1 h at room temperature with anti-HBHA monoclonal antibody VF2, followed by incubation with goat anti-mouse antiserum conjugated to horse radish peroxidase (HRP, Abcam). The blots were revealed with a chemiluminescent substrate of HRP (ECL Prime Western Blotting Detection Reagent, Amersham GE Healthcare) and an Amersham^TM^ Imager 600 (GE Healthcare). Alternatively, lipid-bearing membrane dots were designed using increasing concentrations of lipids, according to Dowler et al. (2002). Briefly, lipids were diluted in the solvent suggested by the manufacturer (usually mix of chloroform and methanol) to reach the desired concentrations. Two-microliter of each lipid sample were spotted in a single row onto an immobilon-P membrane (Millipore). The strips were allowed to air dry for 1 h in the dark. After drying, spotted membranes were incubated and processed as described above.

### Purification of ILI

Intracytosolic lipid inclusions from BCG were isolated as previously described (Low et al., 2010) from bacteria cultured with progressive hypoxia according to the method described by Wayne and Hayes (1996). Briefly, BCG pellets were suspended in 10 mM Tris-HCl (pH 7.5) containing protease inhibitor (complete^TM^, EDTA-free Protease Inhibitor Cocktail, Roche) and lysed by passing four times through a French pressure cell (SLM Aminco) set at 1,240 bar. The lysate was adjusted to 20% final sucrose in 10 mM Tris-HCl (pH 7.5) containing protease inhibitor and overlayed with 10 mM Tris-HCl (pH 7.5) containing protease inhibitor in centrifuge tubes. After 30 min centrifugation at 28,000 × *g* at 4°C in the SW40Ti rotor, the floating layer was harvested and processed for the lipid aggregation assay. Lipid bodies were purified from *R. opacus* PD630 as previously described (Low et al., 2010) with minor modifications. Bacterial cultures grown in liquid medium were harvested by centrifugation at 6,000 × *g* for 20 min, resuspended in 25 ml of 10 mM Tris-HCl (pH 7.5) containing protease inhibitor and lysed by passing three times through a French pressure cell at 1,240 bar. An equal volume of 80% glycerol was added to lysates followed by centrifugation at 5,000 × *g* for 1.5 h at 4°C. The upper layer (lipid body fraction) was then removed and resuspended in 40 ml 40% glycerol, followed by a second centrifugation at 5,000 × *g* for 1.5 h. The upper lipid body layer was harvested and diluted in 20 ml 10 mM Tris-HCl (pH 5) containing protease inhibitor, followed by centrifugation at 50,000 × *g* for 1 h in SW40Ti rotor (Beckman). The pellet was then resuspended in 10 mM Tris-HCl (pH 7.5) containing protease inhibitor. After OD measurement, the lipid solution was used immediately in aggregation test.

### ILI Aggregation Assay

Purified ILI were diluted with 10 mM Tris-HCl (pH 7.5) containing protease inhibitor and distributed in a round-bottom 96-well dish. HBHA (15 μg/well) was added and the plates were incubated at room temperature for 24 h. Lysozyme and BSA (Sigma) were used as positive or negative aggregation controls, respectively. Wells without added protein were included in the assay to check lipid solution. Wells were imaged using a Canon Powershot SD1200 IS digital camera (Canon, Lake Success, NY, United States).

## Results

### HBHA Protein Displays Two Localizations in Mycobacteria

**FIGURE 1:**
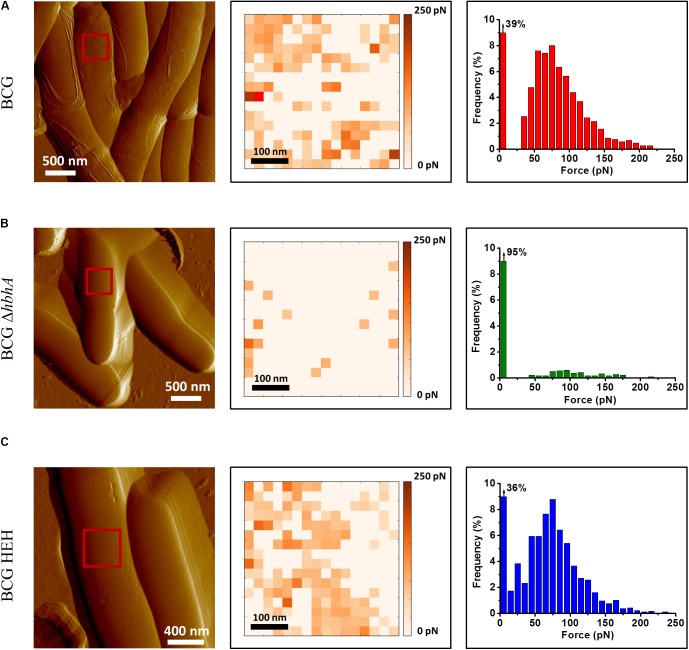
Recombinant strain BCG-HEH display an EGFP-tagged HBHA at the surface that is still able to bind heparin. AFM tomographic image (left panel), representative spatially resolved map of adhesion forces recorded with AFM heparin-coated tip (medium panel) and corresponding histogram (in each case, histogram was obtained from at least 1536 force curves) for **(A)** BCG, **(B)** BCG*ΔhbhA*, and **(C)** BCG-HEH. All the strains were cultured in Sauton’s medium. For each image, the red square corresponds to the area scanned for the determination of one adhesion map. Three independent experiments have been performed.

**FIGURE 2:**
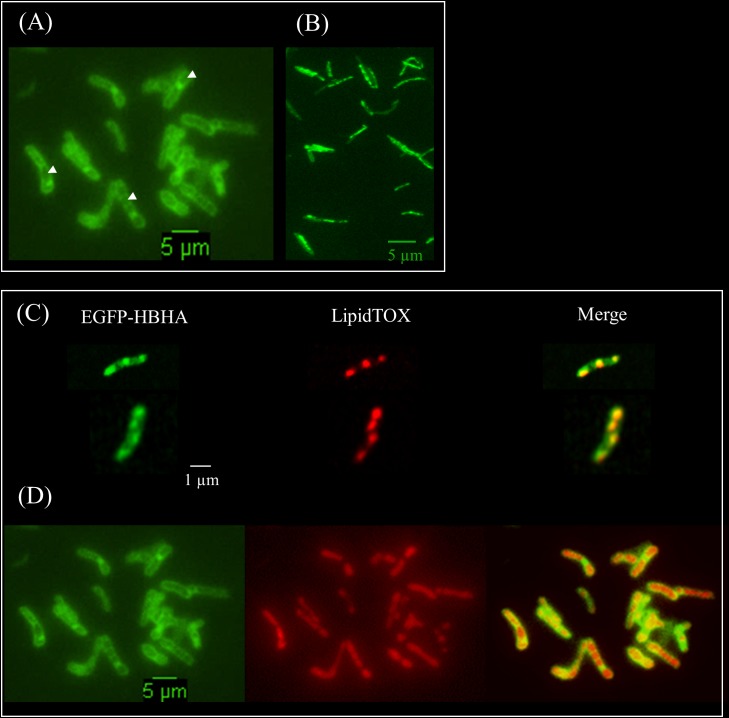
Fluorescent micrographs of EGFP-HBHA hybrid protein and lipid-producing BCG. **(A)** Green fluorescence of EGFP-HBHA-producing BCG observed at the surface and within the cytoplasm (phenotype A). **(B)** Green fluorescence of EGFP-HBHA-producing BCG observed as vesicles in the cytoplasm (phenotype B). **(C,D)** LipidTOX-staining of EGFP-HBHA-producing BCG observed as phenotype B **(C)** or as phenotype A **(D)**. Green fluorescence corresponds to the EGFP-HBHA hybrid protein (left). Red fluorescence is the signal of LipidTOX-stained bacteria (middle), and the merge of both is on the right. Images are representative of four independent experiments. Magnification, ×100.

HBHA has previously been shown by immune-electron microscopy on sections of PFA-fixed bacteria (Menozzi et al., 1996) and by AFM on living cells (Dupres et al., 2005) to be located at the outermost surface of *M. tuberculosis* and *Mycobacterium bovis* BCG. Here, we used fluorescent time-lapse microscopy to investigate the dynamics of HBHA localization on living mycobacteria. A construct coding for a chimeric protein with EGFP fused to the C-terminal part of HBHA, under the control of the *hsp60* promoter, was introduced as a single copy into the chromosome of the HBHA-deficient *M. bovis* strain, BCG*ΔhbhA* (Pethe et al., 2001). The recombinant strain (BCG-HEH) produced an EGFP-tagged HBHA of 48 kDa (**Supplementary Figure S1**) and showed similar growth characteristics than the parental strain. The chimeric fluorescent protein is still able to bind to heparin and is present at the surface of bacteria. Indeed, AFM with a heparin coated tip on living BCG-HEH revealed that the adhesion events frequencies are similar to that obtained with wild-type BCG (**Figures 1A,C**), but clearly higher than those obtained with the HBHA-deleted strain (**Figure 1B**). In agreement with previous studies (Menozzi et al., 1996; Dupres et al., 2005), the tagged protein was seen in fluorescent microscopy at the bacterial surface but also, albeit to a lesser extent, in structures defining compartments within the cytoplasm indicated by white arrows in **Figure 2A** (phenotype A). Surprisingly, in some bacteria a second localization was observed in vesicles aligned all along the cytoplasm (phenotype B, **Figure 2B**). This phenotype resembles the images seen in *M. tuberculosis* stained for TAG-containing ILI (Garton et al., 2002, 2008), suggesting that HBHA may be associated with ILI. To test this hypothesis, the recombinant bacteria were incubated with the lipophilic dye LipidTOX Red, staining the neutral lipids and observed by fluorescence microscopy. The localization of the green vesicles observed in phenotype B coincided with that of red-labeled vesicles (**Figure 2C**). In phenotype A, bright red fluorescence was found within the compartments surrounded by EGFP-HBHA (**Figure 2D**), indicating that these structures could correspond to ILI for TAG storage. This second localization is supported by the recent description of *M. smegmatis* HBHA homolog (MSMEG_0919) as a major protein associated with ILI by proteomic analysis of isolated ILI (Armstrong et al., 2018).

**FIGURE 3:**
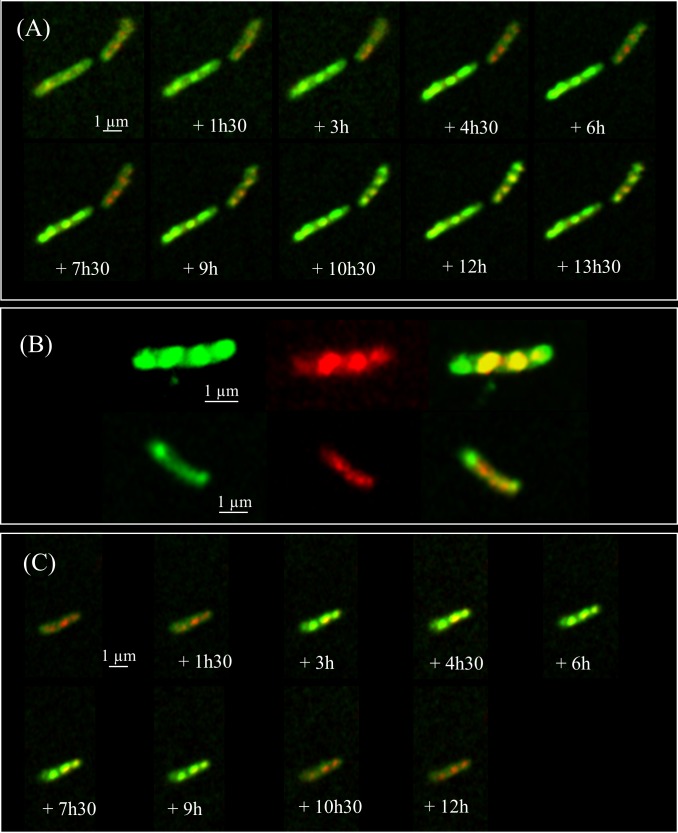
Time-lapse fluorescence micrographs of EGFP-HBHA-producing BCG. Images were captured every 90 min. **(A)** Appearance of phenotype B over time corresponding to EGFP-HBHA localized in vesicles in the cytoplasm. **(B)** Phenotype B of EGFP-HBHA co-localized with the LipidTOX-stained ILI. Green fluorescence is also sometimes seen at the poles in the absence of the red fluorescence signal. **(C)** In some bacteria, phenotype B appeared transiently on the time-lapse snapshots. Green fluorescence corresponds to the EGFP-HBHA hybrid protein. Red fluorescence is the signal of LipidTOX-stained bacteria, and the merge of both is shown in yellow. Results are representative of two independent experiments. Magnification, ×63.

To further understand the relationships between the two phenotypes and ILI, we performed kinetic analyses of the EGFP-HBHA expressing bacteria grown in medium supplemented with LipidTOX Red at a single cell level every 90 min by using time-lapse microscopy. As shown in **Figure 3A**, a dynamic interchange was observed between the two different localizations of EGFP-HBHA during growth. At the beginning, the bacilli displayed green fluorescence at their cell surface (phenotype A). Over time, a switch occurred from phenotype A to phenotype B with strongly labeled intracellular vesicles. The green signal appeared in proximity to or surrounding the red fluorescence (**Figure 3B**). However, in some cases, green spots were seen at the poles of the bacteria without a red signal. For some bacilli, a return from phenotype B to phenotype A was observed (**Figure 3C**). Thus, HBHA-EGFP switches dynamically from one phenotype to the other.

### HBHA Is Involved in the Generation of ILI

**FIGURE 4:**
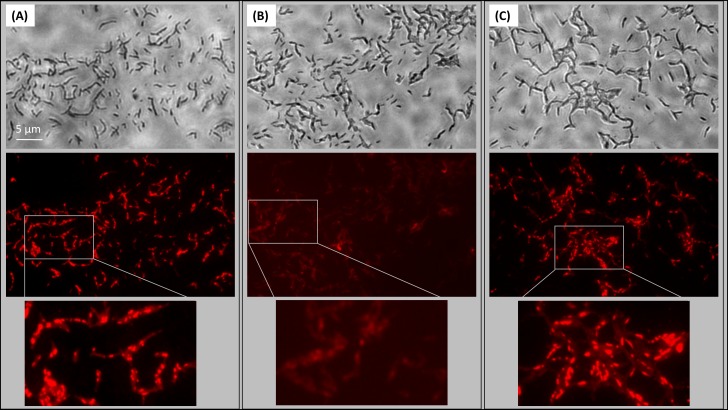
Role of HBHA in ILI formation. Transmission micrograph (upper panels) and fluorescent micrograph (lower panels) of ILI Nile Red-stained in NO-stressed BCG **(A)**, HBHA-deficient BCG*ΔhbhA* **(B)**, and pYUB-HBHA-complemented BCG*ΔhbhA* **(C)**. Images are representative of 14 independent experiments for BCG and BCG*ΔhbhA* and nine independent experiments for complemented strain. Magnification, x63.

Given the association of HBHA-EGFP with ILI, it was of interest to know whether HBHA is involved in the generation of ILI. We therefore subjected BCG and the HBHA-deficient BCG*ΔhbhA* mutant (Pethe et al., 2001) in the early growth phase to nitric oxide (NO) stress, a treatment known to induce the formation of ILI (Daniel et al., 2004), and the fixed bacteria were then stained with the lipophilic fluorophore Nile red. Under these conditions, numerous ILI were found in BCG (**Figure 4A**), whereas the BCG*ΔhbhA* mutant was impaired in its ability to accumulate ILI, as very few droplets were visible within the mutant bacteria (**Figure 4B**). Complementation of the mutant with *hbhA* expressed under the control of its own promoter in pYUB-HBHA (Pethe et al., 2001), restored ILI accumulation to a level similar to that obtained with BCG (**Figure 4C**). Immunoblot analyses previously showed that the complemented strain produced HBHA at a level comparable to that of BCG (Pethe et al., 2001). The recombinant strain, BCG-HEH, restored as well ILI staining when NO-stress, meaning that HBHA-EGFP is able to complement the phenotype in the absence of the nHBHA (**Supplementary Figure S2**).

**FIGURE 5:**
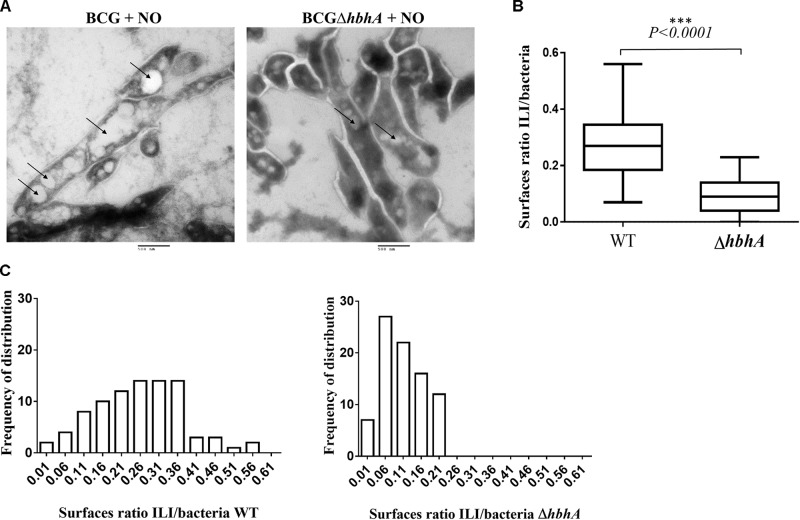
Analysis of ILI content of NO-stressed BCG and BCG*ΔhbhA* by electron microscopy. **(A)** ILI shown as white electron-transparent vesicles (bars = 500 nm), indicated by the arrows. The images are representative of 20 and 15 fields for BCG and BCG*ΔhbhA*, respectively. **(B)** Quantification by ImageJ of ILI surfaces over bacterial surfaces on individual longitudinal sections pictured on TEM fields. The box plots represent the median ratios for each pooled image of the BCG (WT) or BCG*ΔhbhA* (*ΔhbhA*). **(C)** Distribution of ILI surface/bacterial surface ratios for BCG (left panel) and BCG*ΔhbhA* (right panel).

To quantify ILI formation in BCG and BCG*ΔhbhA*, we analyzed the ILI content on sections of NO-stressed bacteria by transmission electron microscopy. As shown in **Figure 5A**, the number of ILI observed as clear white vesicles in the bacteria (black arrows), was higher in BCG than in BCG*ΔhbhA*. To compute precisely this difference, we developed a script in ImageJ^1^ allowing to measure the surface of individual bacteria and the total surfaces of ILI within the bacteria. For BCG, 87 images of longitudinal sections were selected and 967 individual ILI were counted. For BCG*ΔhbhA*, 96 bacteria and 476 individual ILI were measured. The ratio between ILI and bacterial surfaces was markedly reduced in the mutant strain compared to BCG (**Figure 5B**). Moreover, morphometric analyses revealed a higher frequency of ILI of lower sizes in BCG*ΔhbhA* than in BCG (**Figure 5C**), suggesting that HBHA plays a role in the maturation of ILI.

A quantitative comparison of ILI contents of the two strains was also performed by High-Content-Screening (HCS) microscopy. Both GFP-producing BCG (Kremer et al., 1995) and BCG*ΔhbhA* were grown in presence of LipidTOX Red, and the signals for green and red fluorescence were acquired on 6 wells with 16 fields per well. A lower mean intensity of the LipidTOX signal was measured in the mutant compared to the WT (88.9 ± 30.4 and 160.1 ± 54.8 for the BCG*ΔhbhA* and BCG, respectively).

### Induction of ILI in Recombinant *M. smegmatis* Producing BCG HBHA and Role of the Heparin-Binding Site

**FIGURE 6:**
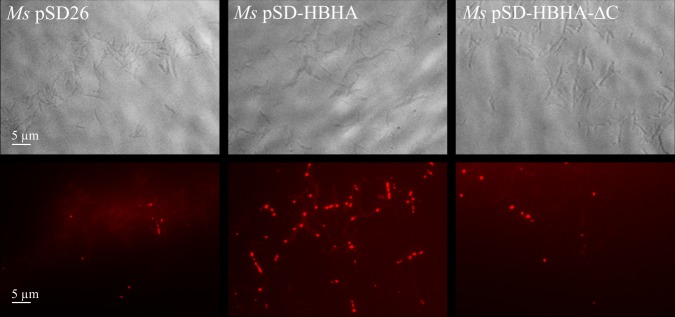
Role of the C-terminal domain of HBHA in ILI formation. Transmission micrograph (upper panels) and fluorescent micrograph (lower panels) of NO-stressed and Nile red-stained *M. smegmatis* (*Ms*) containing pSD26 (left panels), *M. smegmatis* containing pSD-HBHA (middle panels) and *M. smegmatis* containing pSD-HBHA-ΔC (right panels). Images are representative of three independent experiments. Magnification, ×63.

In our hands, *M. smegmatis* produces low amount of ILI in NO-treated cultures, compared to BCG. We thus decided to express BCG-*hbhA* in *M. smegmatis* under the control of an acetamide-inducible promoter (Daugelat et al., 2003) on the pSD-HBHA plasmid and to observe the effect of BCG-HBHA production on the generation of ILI in this recombinant strain. After NO treatment, *M. smegmatis* transformed by the empty vector (pSD26), produced low numbers of ILI as expected (**Figure 6**, left panel). However, when the expression of BCG-*hbhA* was induced in NO-treated *M. smegmatis*::pSD-HBHA, a strong increase of ILI staining was observed (**Figure 6**, middle panel), confirming the involvement of HBHA in lipid storage.

The heparin-binding C-terminal moiety of HBHA, which consists of alanine, proline and methylated lysine repeats, has been shown to be functionally involved in the adherence properties of HBHA to epithelial cells (Pethe et al., 2000). This motif is present, albeit with variable numbers of repeats, in all HBHA-like proteins found in actinomycetes as revealed by BLAST alignments^2^. To evaluate its involvement in the observed effect, we transformed *M. smegmatis* with pSD-HBHA-ΔC, a plasmid coding for HBHA lacking the heparin-binding domain. As shown in **Figure 6** (right panel), truncated HBHA was no longer able to induce ILI accumulation as did the full-length HBHA.

### HBHA Participates in ILI Accumulation in *M. tuberculosis* Within Macrophages

**FIGURE 7:**
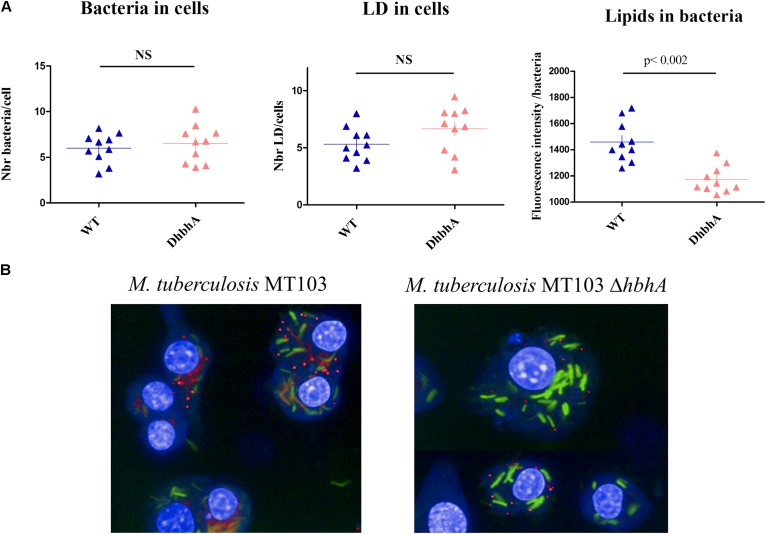
ILI accumulation within *M. tuberculosis* during macrophage infection. RAW264.7 cells were infected by GFP-producing *M. tuberculosis* MT103 (WT) or *M. tuberculosis* MT103*ΔhbhA* (DhbhA), and 24 h later, their nuclei were labeled with DAPI and their lipid content was visualized by LipidTOX labeling. **(A)** Quantification of the numbers of bacteria per cell (left panel), the numbers of cellular lipid droplets per cell (middle panel), and the bacterial ILI content per bacillus (right panel). The data represent image acquisitions in 10 wells on 8–10 fields. NS, not statistically significant **(B)** Images illustrating the accumulation of ILI within *M. tuberculosis* during macrophage infection. Results are representative of two experiments.

Intracellular survival of virulent mycobacteria depends on their ability to induce lipid body formation in macrophages, leading to “foamy” macrophages, devoid of immune activity (Peyron et al., 2008). We thus investigated the possible role of HBHA in both host cell lipid droplets (LDs) and bacterial ILI formation within GFP-producing *M. tuberculosis*-infected mouse macrophages. Analyses performed using the HCS technology showed that WT *M. tuberculosis* (MT103) and HBHA-deficient *M. tuberculosis* (MT103*ΔhbhA*) bacteria were found in murine macrophages at similar levels after 24 h of infection (**Figure 7A**, left panel) and that the deletion of *hbhA* had no effect on cell LDs formation in the infected macrophages (**Figure 7A**, middle panel). In contrast, a significant decrease in mycobacteria ILI was found in HBHA-deficient *M. tuberculosis* compared to HBHA-producing *M. tuberculosis* within infected macrophages (**Figure 7A**, right panel). This difference is illustrated in **Figure 7B**. Thus, HBHA has no effect on LD formation into infected cells, but is involved in ILI accumulation in the bacteria residing within infected macrophages, as observed with free mycobacteria in *in vitro* cultures.

### HBHA-Mediated Aggregation of ILI

**FIGURE 8:**
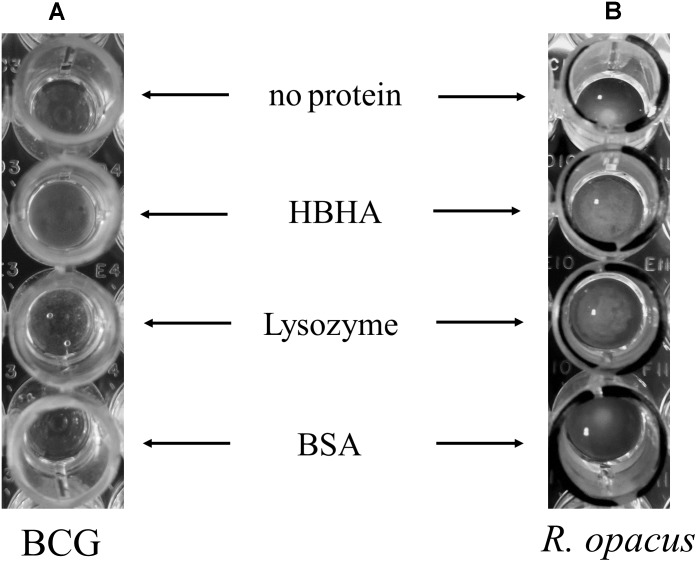
Aggregation of BCG and *R. opacus* ILI by HBHA. ILI purified from **(A)** BCG (150 μl of ILI solutions at OD_600nm_ = 0.1 corresponding to 220 ml culture of BCG in hypoxia) or from **(B)** *R. opacus* (20 μl of ILI solution at OD_600nm_ = 3 corresponding to a 22 ml culture of *R. opacus*) were mixed in a 180 μl final volume with 15 μg of purified HBHA, commercial Lysozyme or BSA, or without any added protein, as indicated, and deposited in wells of round-bottom 96-well plates. After 24 h incubation at room temperature, the ILI aggregation was examined by visual inspection.

To further study the interaction of HBHA with ILI, we purified BCG ILI from hypoxia-stressed bacteria and incubated them *in vitro* with purified HBHA in round-bottom 96-well dishes. Control proteins used were lysozyme, known to aggregate lipids (Al Kayal et al., 2012) and BSA, as negative control. As shown in **Figure 8A**, some aggregation of BCG ILI occurred after incubation with HBHA. However, this aggregation appears relatively weak due to the difficulty to obtain large amounts of ILI from low-growing BCG (see legend of the figure). We thus decided to repeat this experiment with other ILI easily isolated from *R. opacus* PD630, an actinobacteria known as a high lipid (TAG) producing bacteria. Interestingly, this organism produces TadA protein, a homolog of HBHA (MacEachran et al., 2010), which is involved in lipid accumulation and which strongly aggregated homologous ILI. Results presented in **Figure 8B** revealed a marked aggregation of *R. opacus* ILI with HBHA, or with the positive control protein, but not with BSA, confirming and reinforcing the result on capacity of HBHA to aggregate ILI. This also indicates that HBHA can directly interact with ILI in the absence of any additional mycobacterial protein.

### HBHA Interacts With Phosphoinositides

**FIGURE 9:**
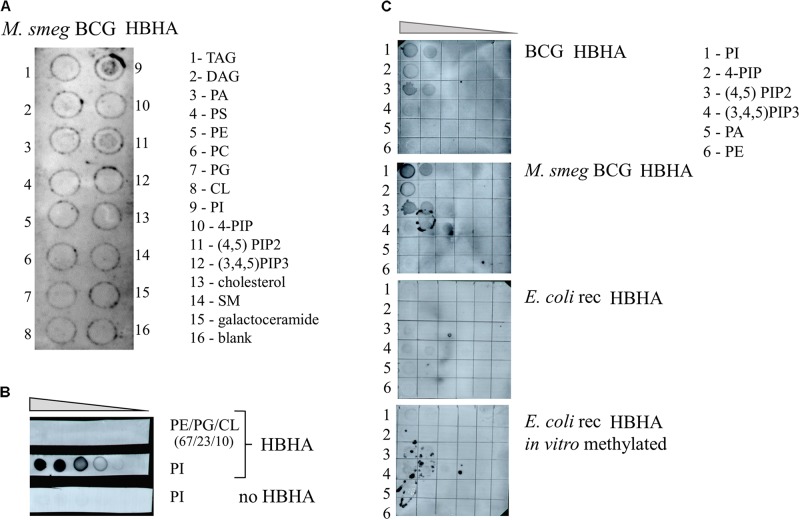
HBHA Lipid-binding assay. **(A)** Commercial membrane lipid strips were incubated with HBHA purified from recombinant *M. smegmatis* producing BCG HBHA. **(B)** *E. coli* polar lipid extract (PE/PG/CL at ratio 67/23/10) or phosphatidylinositol (PI) was spotted onto membranes at concentrations varying from to 1 μg to 62.5 ng and incubated with or without HBHA, as indicated. **(C)** Indicated lipid species were spotted onto membranes at concentrations ranging from 250 to 16 ng and membranes were incubated with nHBHA (upper panel), BCG HBHA produced by recombinant *M. smegmatis* (upper middle panel), rHBHA (lower middle panel), or *in vitro*-labeled rHBHA (lower panel). Lipid binding was revealed using the anti-HBHA monoclonal antibody VF2. TAG, Glyceryl tripalmitate; DAG, Diacylglycerol; PA, phosphatidic acid; PS, Phosphatidylserine; PE, Phosphatidylethanolamine; PC, Phosphatidylcholine; PG, Phosphatidylglycerol; CL, cardiolipin; PI, Phosphatidylinositol; 4-PIP, Phosphatidylinositol 4-phosphate; (4,5)PIP2, Phosphatidylinositol 4,5-bisphosphate; (3,4,5)PIP3, Phosphatidylinositol (3,4,5)-triphosphate; and SM, sphingomyelin.

To investigate whether HBHA can directly interact with lipids and to determine the nature of such lipids, we first screened arrays of 15 lipids using the protein-lipid overlay (PLO) assay as supplied by Echelon Biosciences. As no large pattern of lipids from bacterial origin was commercially available, we used purified lipids from eukaryotes for the analysis. This lipid strip contains spots of the major lipid components of membranes, such as phosphatidylcholine, phosphatidylethanolamine, cardiolipin, and phosphoinositides (PIP), but also triglyceride (TAG), the major component of ILI. The membrane was first incubated with BCG HBHA produced by recombinant *M. smegmatis*, and the lipid-binding capability of HBHA was revealed using the monoclonal anti-HBHA antibody VF2. This initial screening identified phosphatidylinositol (PI) and 4,5 di-phosphorylated PI (PIP2) as HBHA-binding lipids (**Figure 9A**). No binding was observed to the other lipids, including TAG.

Next, increasing amounts of PI or *E. coli* lipid extract, which did not contain this lipid, were spotted onto membranes and probed with HBHA purified from BCG and revealed by the monoclonal antibody VF2. Only the combination of PI and HBHA gave rise to a positive signal (**Figure 9B**), no signal being observed with PI in the absence of HBHA, or with the *E. coli* extract incubated with HBHA.

We then spotted different species and amounts of PI on the membranes and incubated them with either nHBHA isolated from BCG, BCG-HBHA produced by recombinant *M. smegmatis*, recombinant HBHA produced in *E. coli* (rHBHA) or *in vitro* methylated rHBHA. As shown in **Figure 9C**, nHBHA and recombinant HBHA produced in *M. smegmatis* bound to PI, and PIP2, but barely to mono or tri-phosphorylated PI. Almost no binding was observed for non-methylated rHBHA produced in *E. coli*, or *in vitro* methylated rHBHA. These observations not only show that the methylation of HBHA is essential for its interaction with lipids, but also that a precise methylation pattern of the heparin-binding C-terminal domain of HBHA is required.

## Discussion

Using fluorescence microscopy, high content screening and electron microscopy, we show here that HBHA is involved in the formation and accumulation of ILI, both in BCG cultured *in vitro* and in *M. tuberculosis* residing within infected macrophages. When compared to BCG, HBHA-deficient BCG*ΔhbhA* displayed significantly lower numbers of ILI and their sizes were smaller. Nevertheless, the mutant strain still contained some ILI, indicating that HBHA has a major role but is not absolutely required for ILI formation. The association of HBHA with ILI was evidenced by the co-localization of EGFP-tagged HBHA produced ectopically in BCG*ΔhbhA* with LipidTOX Red-labeled ILI using fluorescence microscopy. As expected from previous studies, HBHA-EGFP was also observed at the surface of the bacilli, by AFM and fluorescence microscopy, in addition to its presence surrounding lipid vesicles. A dynamic interchange between these localizations was detected by time-lapse microscopy of growing HBHA-EGFP-producing BCG. Images captured every 90 min on single bacteria showed the presence of HBHA-EGFP at the surface of BCG, which then moved into the cytosol to form spots distributed along the rod-shaped bacilli, before returning to the initial localization. In most of the observed bacteria, these spots were co-localized with ILI. In some cases, they were not associated with ILI, but were present at the poles of growing bacteria. Moreover, AFM studies on the recombinant strain producing HBHA-EGFP showed that this chimeric protein at the bacterial surface is still able to bind heparin, signifying that EGFP grafting do not interfere with its biogenesis and functionality.

Together, these observations suggest that HBHA plays a structural role in the biogenesis of ILI, potentially participating in a network of proteins surrounding lipids and migrating from the plasmatic membrane toward the boundary layer of ILI. Little is known about the molecular mechanisms involved in the formation of TAG inclusions in prokaryotes, although enzymes involved in TAG synthesis have been identified in *M. tuberculosis* (Kalscheuer and Steinbuchel, 2003; Daniel et al., 2004; Sirakova et al., 2006; Elamin et al., 2011). The production of TAG is limited to actinomycetes, a small number of Gram-negative bacteria and certain cyanobacteria (Alvarez and Steinbuchel, 2002; Daniel et al., 2004, 2011) and, interestingly, HBHA-related proteins were only found in actinobacteria. The HBHA homolog TadA of *R. opacus* PD630 was found to be associated with ILI in these oleaginous bacteria and to regulate ILI assembly and/or maturation both *in vivo* and *in vitro* (MacEachran et al., 2010). Furthermore, the expression of *tadA* was increased when *R. opacus* was cultured in a low nitrogen/carbon ratio medium (Chen et al., 2014). Proteomic analyses of *Rhodococcus sp.* RHA1 ILI indicated that MLDS (ro02104), a TadA homolog, is one of the two major ILI-associated proteins (Ding et al., 2012).

In both *Rhodococcus* species, mutants lacking the HBHA homolog presented ILI of increased size, although the TAG content was decreased in TadA mutant. In contrast, we observed a decrease in size of ILI in the absence of HBHA. The size of ILI depends on an equilibrium between storage and utilization of TAG. The discrepancies observed between *Rhodococcus* and *Mycobacterium* could be due to different turnover rates in the biosynthesis and catabolism of TAG between these two species which are, respectively, fast and slow-growing bacteria. However, MacEachran et al. (2010) observed a defect in the coalescence of ILI at later time points in the absence of TadA. Lanfranconi and Alvarez (2016) proposed that TadA and HBHA have evolved differently in *Rhodococcus* and *Mycobacterium* giving rise to functional divergence between the two proteins. Our data do not support this hypothesis and show that, in addition to its adhesin properties, HBHA has conserved a role in ILI accumulation and maturation. Recently, the *M. smegmatis* HBHA homolog (MSMEG_0919) has been found as the major ILI-associated protein (Armstrong et al., 2018).

Lipid droplets are ubiquitous and evolutionarily conserved organelles found in almost all organisms, from bacteria to mammals. In eukaryotes, knowledge and understanding of their dynamics and regulation are growing exponentially these last years and demonstrated a crucial role of lipids droplets far beyond simply a lipid storage organelle. Formation of LDs requires many different enzymes and structural proteins from the budding of the vesicles to their maturation, fusion, fission, and regulation of their content. Positioning of LDs into the cytoplasm and transport of lipid cargo is the result of interactions between these proteins and motor proteins or cytoskeletal elements. Phospholipid monolayer of nascent LDs is derived from the endoplasmic reticulum membranes; however, little is known about addition of phospholipids to maturing LDs. This could occur either by diffusion of lipids into the LDs close to endoplasmic reticulum or by trafficking through the cytosol on phospholipid transfer proteins (Brasaemle and Wolins, 2012).

In prokaryotes, it is thought that ILI are delimited by a phospholipid monolayer, by analogy with eukaryotes. *R. opacus* PD630 ILI have indeed been shown in electron microscopy to be surrounded by a thin boundary layer that could correspond to a monolayer membrane (Alvarez and Steinbuchel, 2002), although it was not already shown for mycobacteria. Using commercially available lipids, we found that HBHA may interact *in vitro* with eukaryote PIPs, in particular with its bi-phosphorylated form PI(4,5)P2 (PIP2). In eukaryotic cells, PIPs represent only a small fraction of membrane lipids but appear to play a major role in cell signaling and in organelle biology, ranging from vesicular trafficking to transport of proteins across membranes (Balla et al., 2009; Balla, 2013). PIPs correspond to phosphorylated forms of phosphatidylinositol, and seven different PIPs have been identified differing by the phosphorylation pattern of the inositol ring. Each phosphorylated form of PIPs present a specific distribution in cellular organelles (Picas et al., 2016). PIP2 has been identified as a signature of the plasma membrane and, more recently, of LDs (Li et al., 2008). It is also involved in the budding of the plasma membrane necessary for LD formation. In contrast to eukaryotes, which are all able to synthetize phosphatidylinositol, only few bacteria contain phosphatidylinositol but actinobacteria do produce phosphatidylinositol. It is thus tempting to envisage an interaction of HBHA with mycobacterial PIP-like lipids that could lead to their transport from the bacterial cell wall toward intracellular ILI. The interaction we observed *in vitro* between HBHA and eukaryote PIP2 could in fact reflect an interaction of HBHA with a species of bacterial lipid either not yet identified or already known and structurally closed to this PIP. Until now, only one phosphoinositide, PIP3, was shown to be *de novo* synthetized in *M. smegmatis* in stress response (Morita et al., 2010). In mycobacteria, phosphatidylinositol is the precursor of lipophosphoglycans, such as phosphatidylinositol mannosides or lipoarabinomannan, two major constituents of the mycobacterial cell wall (Torrelles et al., 2006; Morita et al., 2011). Moreover, some mycobacterial phospholipids such as PIM1 and PIM2 presents structural mimicry with eukaryote phosphoinositides, with the mannose residues replacing the phosphate groups (Vergne et al., 2004; Alix et al., 2011).

Heparin-binding hemagglutinin adhesin is an IDP (Lebrun et al., 2012). Therefore, a specific function could not be deduced for this protein from its amino-acid sequence. IDP proteins adopt a defined structure only after binding to a specific ligand. Submitting HBHA to structure prediction algorithms (Zhang, 2008) identified apolipoproteins and proteins with BAR domains participating in membrane curvature (Peter et al., 2004) by homologies of the coiled-coil domains. Hidden Markov Model alignment of a region covering the coiled-coil domain of HBHA sequence revealed apolipoprotein A1/A4/E domain as the highest significant match (**Supplementary Figure S3**). Apolipoprotein-A1 has been shown to bind PIPs, including PIP2, and is able to complex them by dimerization, thereby allowing lipid efflux from the cells (Gulshan et al., 2016). It is therefore plausible that HBHA interacts with PIP-like lipids in a similar fashion leading to different localizations of HBHA in the mycobacteria as described here. Apolipoprotein-like HBHA could act as a cargo to transport PIPs-like lipids from the plasma membrane toward ILI. This refueling is suggested by time-lapse images showing the movement of EGFP-HBHA from the plasma membrane toward ILI and back. In some time-lapse images, a polar HBHA accumulation was observed during the growth of bacteria in spots that did not contained neutral lipids (**Figure 3B**). This may potentially correspond to an important need of phospholipids necessary for elongation of the bacterial membrane (Thanky et al., 2007).

Although these observations suggest that the coiled-coil domain of HBHA is crucial for PIPs-like lipids transport to ILI during their development, we found that the lysine-rich C-terminal domain of HBHA is also essential for both *in vitro* lipid binding and ILI formation. Only methylated nHBHA or recombinant HBHA produced in *M. smegmatis* were able to bind PI and PIP2. Non-methylated rHBHA produced by *E. coli*, or *in vitro*-methylated rHBHA did not bind to these lipids, indicating that both the C-terminal domain and its proper methylation pattern are critical for the interaction between HBHA and lipids. Obviously, the positively charged C-terminal domain has the potential to bind negatively charged phospholipids by electrostatic interactions, however, this is clearly not sufficient for HBHA binding to PIP2. It may therefore be possible that the methylation of the lysines may facilitate and/or stabilize the binding of intrinsically disordered HBHA to its ligand, possibly by increasing hydrophobicity of HBHA and thus its interaction with the hydrophobic moiety of the lipids (Parish and Rando, 1996).

Mycobacteria store TAGs in the form of ILI during hypoxia leading to non-replicating persistence (Garton et al., 2002; Daniel et al., 2004). Importantly, ILI-positive acid-fast bacilli found in sputum were considered as markers for non-replicating *M. tuberculosis* cells during human infection (Garton et al., 2008; Low et al., 2010). These lipid-storing bacteria are phenotypically drug-resistant and probably the cause for prolonged TB treatment required for therapy (Low et al., 2010). HBHA has been defined as a latency antigen (Hougardy et al., 2007) and is shown in this study to be involved in the accumulation of ILI by the mycobacteria, which may be connected. The fact that this protein is also an important adhesin for *M. tuberculosis* binding to non-phagocytic cells and that, recently, its homolog MLDS was shown to bind DNA to the ILI (Zhang et al., 2017) may qualify HBHA as a moonlighting protein serving several functions depending on its location and its ligands.

## Author Contributions

DR and CV conceived, designed, and performed the experiments. BC, ML, CR, EW, GD, and PB contributed to materials and analysis tools. DR, CV, GD, and EW performed the data analysis. DR, CV, and CL wrote the paper.

## Conflict of Interest Statement

The authors declare that the research was conducted in the absence of any commercial or financial relationships that could be construed as a potential conflict of interest.

## Footnotes

1 https://imagej.nih.gov/ij/

2 https://blast-ncbi-nlm-nih-gov.gate2.inist.fr
