# Supplementary material for: Heparin-Binding Hemagglutinin Adhesin (HBHA) Is Involved in Intracytosolic Lipid Inclusions Formation in Mycobacteria
Source: Front Microbiol. 2018 Sep 24;9:2258. doi: 10.3389/fmicb.2018.02258 (PMC6176652; doi:10.3389/fmicb.2018.02258)
Supplement: Supplementary file 1 [file Data_Sheet_1.docx]

Supplementary Material

Heparin-binding Hemagglutinin Adhesin (HBHA) is involved in intracytosolic lipid inclusions formation in mycobacteria

**Dominique Raze1*$, Claudie Verwaerde1$, Gaspard Deloison1, Elisabeth Werkmeister1, Baptiste Coupin1, Marc Loyens1, Priscille Brodin1, Carine Rouanet1, and Camille Locht1***

**Correspondence:** Corresponding Author: [dominique.raze@ibl.cnrs.fr](mailto:dominique.raze@ibl.cnrs.fr)

Table S1: list and origin of strains, plasmids and primers

| **Strains, plasmids or primers** | **Genotype or description** | **Reference** |
| --- | --- | --- |
| Strains  *E. coli* |  |  |
| Xl1Blue | recA1 endA1 gyrA96 thi-1 hsdR17 supE44 relA1 lac [F´ proAB lacIqZ∆M15 Tn10 (Tetr)]. | Lab collection |
| DH5α | F- φ80lacZΔM15 Δ(lacZYA-argF) U169 recA1 endA1 hsdR17 (rk-, mk+) phoA supE44 λ- thi-1gyrA96 relA1 | Lab collection |
| *Mycobacterium* |  |  |
| MT103 | Laboratory strain *M. tuberculosis* MT103 | (Jackson et al., 1999) |
| MT103Δ*hbhA* | *M. tuberculosis* Mt103 deleted of *hbhA* | (Pethe et al., 2001) |
| MT103-GFP | *M. tuberculosis* Mt103::pGFM11 | This study |
| MT103Δ*hbhA*-GFP | *M. tuberculosis* Mt103 Δ*hbhA*::pGFM11 | This study |
| BCG | Laboratory strain *M. bovis* BCG1173P2 |  |
| BCGΔ*hbhA* | *M. bovis* BCG1173P2 deleted of *hbhA* | (Pethe et al., 2001) |
| BCG-GFP | *M. bovis* BCG1173P2::pGFM11 | This study |
| BCGΔ*hbhA*-GFP | *M. bovis* BCG1173P2 Δ*hbhA*::pGFM11 | This study |
| BCG-HEH | *M. bovis* BCG1173P2 deleted of *hbhA* :: pMV361-HEH |  |
| *M. smegmatis* mc^2^155 | Laboratory strain *M. smegmatis* mc^2^155 | ATCC 700084 |
| *Ms* pSD26 | *M. smegmatis* mc^2^155 :: pSD26 | This study |
| *Ms* pSD-HBHA | *M. smegmatis* mc^2^155 :: pSD-HBHA | This study |
| *Ms*-pSD-HBHA-ΔC | *M. smegmatis* mc^2^155 :: pSD-HBHA-ΔC | This study |
| *Rhodococcus* |  |  |
| *R. opacus* PD630 | DSM 44193 | (Alvarez et al., 1996) |
| Plasmids |  |  |
| pYUB-HBHA | *hbhA* with its promoter in pYUB415 | (Pethe et al., 2001) |
| pGFM-11 | *gfp*-expressing shuttle vector | (Kremer et al., 1995) |
| pSD26 | Shuttle vector with acetamidase inducible promoter | (Daugelat et al., 2003) |
| pSD-HBHA | *hbhA* inserted in pSD26 | This study |
| pSD-HBHA-ΔC | *hbhA* deleted of C-terminal heparin-binding domain inserted in pSD26 | This study |
| pMV361 | Integration vector for expression under *hsp60* promoter | (Stover et al., 1991) |
| pMV361-HEH | pMV361 *egfp* fused to C-terminal end of *hbhA* | This study |
| Primers |  |  |
| SPhbhA pSD | GGATCCGCTGAAAACTCGAACATTGA |  |
| ASPhbhA pSD | GATATCCTACTTCTGGGTGACCTTCTTGG |  |
| ASPhbhADC pSD | GATATCCTACAGCTCGATGCCGACCAGCTTGG |  |
| SPhbhA Sph Bam | gcatgcaagctcaggattcgaccg |  |
| ASPhbhA Sph Bam | GGATCCCTTCTGGGTGACCTTCTTGG |  |
| SP/PCR2 egfp1 | ggatccgtgagcaagggcga |  |
| ASP/PCR2 egfp1 | TTAATTAATCACTTGTACAGC |  |
| SPHE | gaattccatggctgaaaactcgaaca |  |
| ASPHE | AAGCTTTAATCACTTGTACAGCTCGTC |  |
| SP hygR | gctagcgcatgccacctagatccttt |  |
| ASP hygR | ACTAGTAGCTAGAGGGGCGTCAGGCG |  |


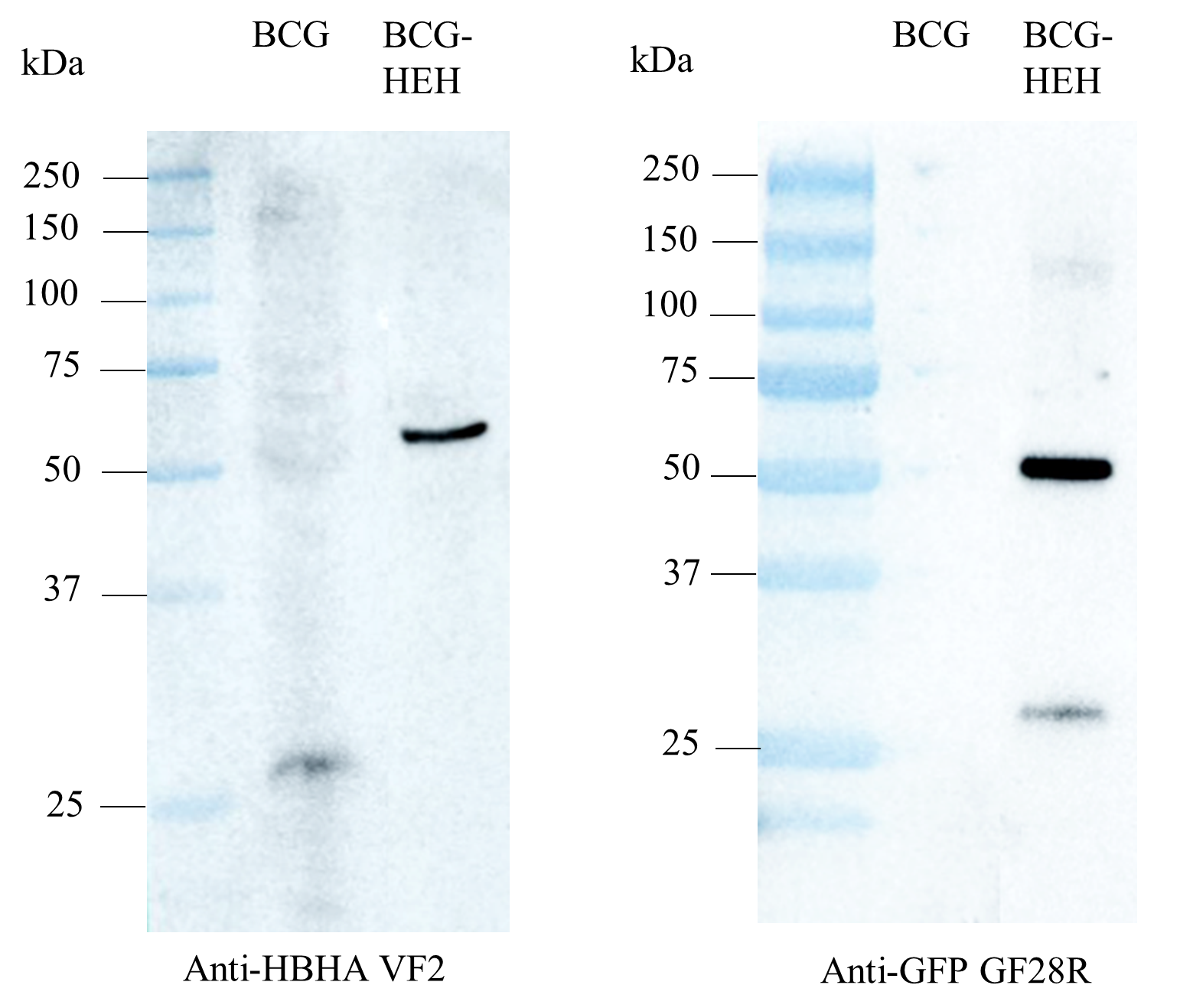


Figure S1. Immunoblot analyses of the protein content of BCG or recombinant BCG producing the EGFP-HBHA hybrid protein (BCG-HEH) using either anti-HBHA monoclonal antibody VF2 (left panel) or anti-GFP monoclonal antibody GF28R (right panel).


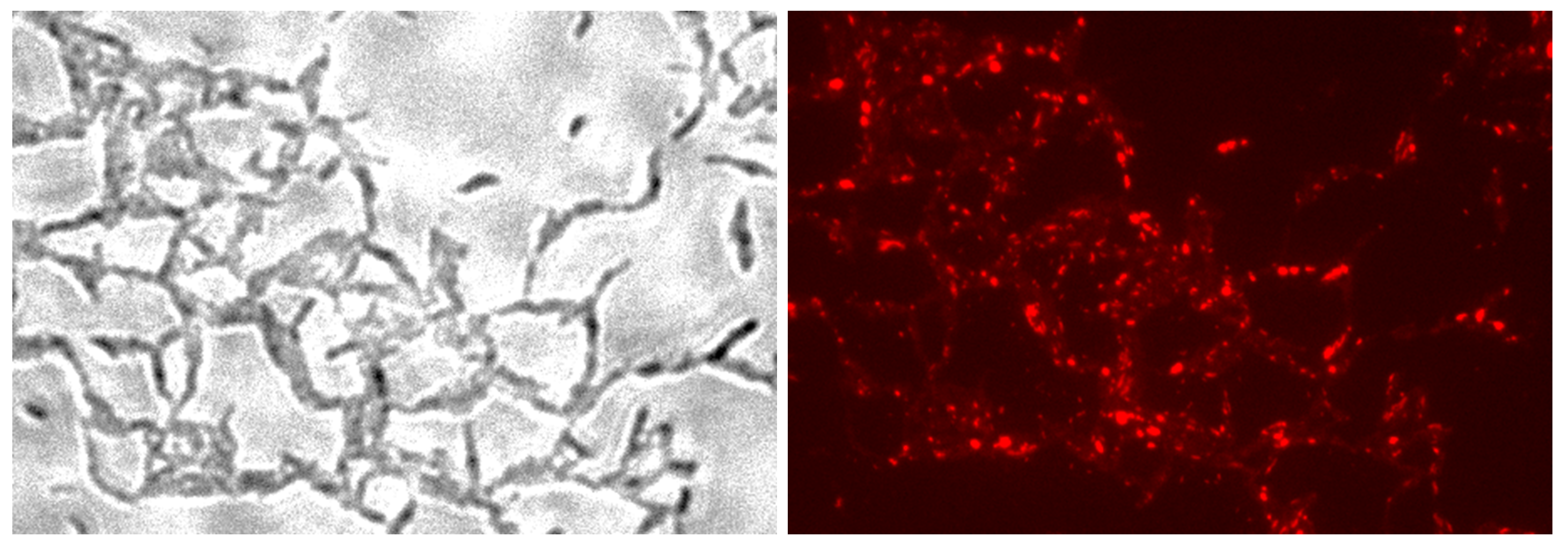


Figure S2. Role of HBHA-EGFP in ILI formation. Transmission micrograph (left panel) and fluorescent micrograph (right panel) of ILI Nile Red-stained in NO-stressed BCG-HEH. Images are representative of four independent experiments. Magnification, x63

Figure S3. HBHA structure prediction and Hidden Markov Model alignment. We subjected HBHA to a structure prediction algorithm by using the I-TASSER server (Zhang, 2008) to compare HBHA to structures of the 3D protein database ([www.rcsb.org](file:///D:\Documents\Papiers\HBHA\Vésicules%20lipidiques-HBHA\Soumission%20mBio\www.rcsb.org)). Among the top ten threading templates found by I-TASSER to construct *ab initio* models, three belong to the apolipoprotein family. The most relevant model was then used to align HBHA with the 3D structures of the PDB database. This alignment revealed apolipoproteins and proteins with BAR domains participating in membrane curvature (Peter et al., 2004) by homologies of the coiled-coil domains. Moreover, hidden Markov Models alignment of a region covering the coiled-coil domain of HBHA sequence (residues 20 to 155) with the Pfam database (<http://pfam.xfam.org>) indicated the apolipoprotein A1/A4/E domain (accession: PF01442.17) as the highest significant match with a degree of confidence for each individual aligned residue ranging from 30 to 80 % (Fig. S3A). By aligning to the Superfamily database (<http://supfam.org>), it revealed the apolipoprotein AI superfamily (accession: 58113) as the highest significant match with a degree of confidence for each individual aligned residue ranging from 50 to 90 % (Fig. S3B).


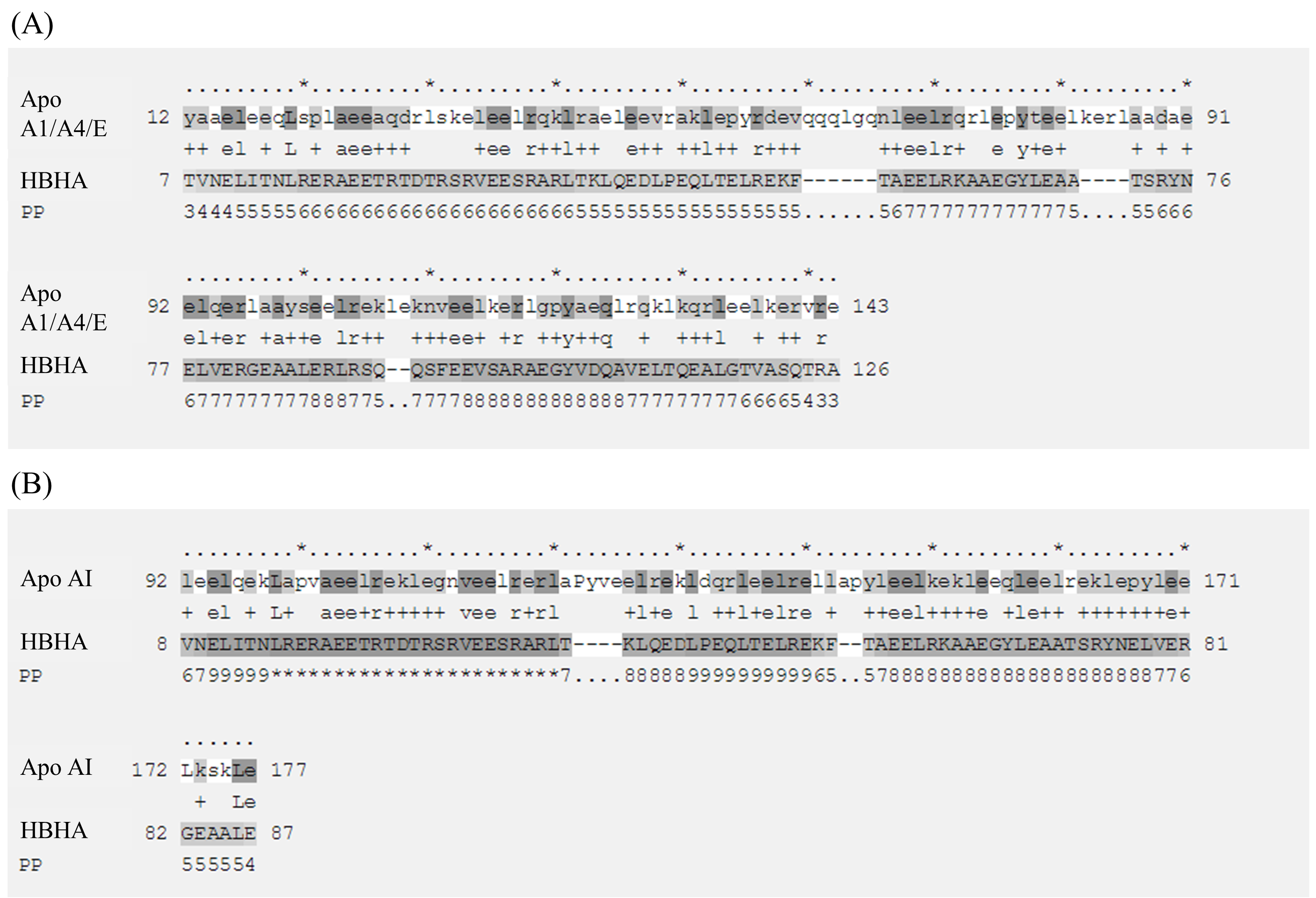


Hidden Markov Model alignment between the coiled-coil domain of HBHA and the Apolipoprotein A1/A4/E Pfam domain (A) or the Apolipoprotein AI superfamilly domain (B). The upper sequence is the model sequence coloured according to the match to query sequence: dark grey for identical residues and light grey for similar residues. In the match line, a “+” sign indicate similar residues matches. The lower sequence in capital letters is the query sequence, coloured according to the posterior probability, the higher it is, the darker the grey. The pp line indicate the posterior probability or the degree of confidence in each individual aligned residue. A 0 means 0-5%, 1 means 5-15%, and so on; 9 means 85-95%, and a * means 95-100% posterior probability.

**Supplementary material references**

Alvarez, H.M., Mayer, F., Fabritius, D., and Steinbuchel, A. (1996). Formation of intracytoplasmic lipid inclusions by Rhodococcus opacus strain PD630. *Arch Microbiol* 165(6)**,** 377-386.

Daugelat, S., Kowall, J., Mattow, J., Bumann, D., Winter, R., Hurwitz, R., et al. (2003). The RD1 proteins of Mycobacterium tuberculosis: expression in Mycobacterium smegmatis and biochemical characterization. *Microbes Infect* 5(12)**,** 1082-1095.

Jackson, M., Phalen, S.W., Lagranderie, M., Ensergueix, D., Chavarot, P., Marchal, G., et al. (1999). Persistence and protective efficacy of a Mycobacterium tuberculosis auxotroph vaccine. *Infect Immun* 67(6)**,** 2867-2873.

Kremer, L., Baulard, A., Estaquier, J., Poulain-Godefroy, O., and Locht, C. (1995). Green fluorescent protein as a new expression marker in mycobacteria. *Mol Microbiol* 17(5)**,** 913-922.

Peter, B.J., Kent, H.M., Mills, I.G., Vallis, Y., Butler, P.J., Evans, P.R., et al. (2004). BAR domains as sensors of membrane curvature: the amphiphysin BAR structure. *Science* 303(5657)**,** 495-499. doi: 10.1126/science.1092586.

Pethe, K., Alonso, S., Biet, F., Delogu, G., Brennan, M.J., Locht, C., et al. (2001). The heparin-binding haemagglutinin of M. tuberculosis is required for extrapulmonary dissemination. *Nature* 412(6843)**,** 190-194. doi: 10.1038/35084083.

Stover, C.K., de la Cruz, V.F., Fuerst, T.R., Burlein, J.E., Benson, L.A., Bennett, L.T., et al. (1991). New use of BCG for recombinant vaccines. *Nature* 351(6326)**,** 456-460. doi: 10.1038/351456a0.

Zhang, Y. (2008). I-TASSER server for protein 3D structure prediction. *BMC Bioinformatics* 9**,** 40. doi: 10.1186/1471-2105-9-40.
